# Association of Serum Uric Acid Status With Bone Mineral Density in Adolescents Aged 12–19 Years

**DOI:** 10.3389/fmed.2020.00255

**Published:** 2020-06-09

**Authors:** Kaiyu Pan, Xiaocong Yao, Minbo Liu, Zhongxin Zhu

**Affiliations:** ^1^Department of Paediatrics, The First People's Hospital of Xiaoshan District, Hangzhou, China; ^2^Department of Osteoporosis Care and Control, The First People's Hospital of Xiaoshan District, Hangzhou, China; ^3^Institute of Orthopaedics and Traumatology, Zhejiang Chinese Medical University, Hangzhou, China

**Keywords:** uric acid, bone mineral density, adolescent, NHANES, cross-sectional study

## Abstract

**Aims:** Evidence linking serum uric acid (sUA) and bone mineral density (BMD) in adolescents is very limited. To the best of our knowledge, only one report has focused on the relationship between sUA and BMD in adolescents. Thus, this study aimed to determine the association between sUA and total BMD in adolescents aged 12–19 years.

**Methods:** A cross-sectional study was conducted on a sample composed of non-institutionalized US population from the National Health and Nutrition Examination Survey. Weighted multivariate linear regression models were used to evaluate the association between sUA and total BMD. Subgroup analyses were further performed.

**Results:** sUA positively correlated with total BMD in the multiple regression model after adjusting for potential confounders. However, in the subgroup analyses stratified by sex, age, or race/ethnicity, the association between sUA and total BMD followed an inverted U-shaped curve in female adolescents, adolescents aged 16–19 years, and other race/ethnicity.

**Conclusions:** Our results suggested that the correlation between sUA level and total BMD differed by sex. The increased sUA level would be beneficial to bone health in adolescents with low sUA levels, but for female adolescents, a higher sUA level (turning point, 3.9 mg/dL) may have an adverse effect on bone health.

## Introduction

During adolescence, bone accumulates and grows at a rapid rate. Bone mineral density (BMD) acquisition during this period is critical for bone mass accrual and skeletal formation to acquire a greater peak bone mass and therefore prevent osteoporosis at older age (1, 2). Therefore, osteoporosis is also recognized as a pediatric disease.

Clinical evaluation of osteoporosis-associated risk factors contributes to early diagnosis, prevention, and management of osteoporosis. Consequently, on-going studies are assessing the correlation of bone health with some less studied or novel biomarkers, such as serum uric acid (sUA).

Convincing experimental studies reported that antioxidants could contribute to the reduction of osteoclast activity and the activation of osteoblasts (3, 4). Conversely, reactive oxygen species could reduce the bone formation activity of osteoblasts and simultaneously stimulate the resorption activity of osteoclasts (5, 6). sUA is the most abundant non-enzymatic endogen antioxidant present in the systemic circulation and had long been recognized as a biologically inert waste product of purine metabolism; however, diagnosis of hyperuricemia is a concern because it causes gout and chronic inflammatory arthritis (7). Moreover, increasing evidence supported that higher sUA level might be beneficial for bone metabolism owing to its antioxidant properties (8).

Recent epidemiological studies reported a positive association between sUA and BMD in middle-aged and older individuals (9–15). Until now, evidence linking sUA and BMD in adolescents is very limited. To the best of our knowledge, only one report has focused on the relationship between sUA and BMD in adolescents, and the result of that cross-sectional study from Iran (221 girls and 192 boys) demonstrated a higher bone density in those who had higher UA levels (16). The prevalence of hyperuricemia in children and adolescents varies among countries, and largely depends on age, sex, ethnicity, and region (17). In addition, concentration of UA is strongly influenced by age, sex, body mass index (BMI), body composition, and other factors that also influence BMD (10, 18, 19). Therefore, based on the national population, we performed a cross-sectional study with a much larger sample to determine the association of sUA with BMD in adolescents.

## Materials and Methods

### Study Population

In this study, data analyzed were obtained from the National Health and Nutrition Examination Survey (NHANES) (1999–2006), a complex, stratified, multistage probability sample of the non-institutionalized US population. These cross-sectional surveys are conducted by the National Center for Health Statistics (NCHS). Methodological details about the NHANES are available at www.cdc.gov/nchs/nhanes/.

The study population was limited to participants aged 12–19 years (*n* = 9,493) with complete data on sUA and total BMD. After exclusion of 2,173 subjects with missing sUA (*n* = 1,184) or total BMD (*n* = 989) data, 7,320 subjects aged 12–19 years remained for the final analysis.

The NCHS Ethics Review Board granted approval for the conduct of NHANES, and written informed consents were obtained from all participants (20). For participants aged <18 years, their parents/guardians provided informed consent, and participants aged ≥18 years provided informed consent on their own.

### Study Variables

The principal variables of this study were sUA (independent variable) and total BMD (dependent variable). SUA levels were measured using a Hitachi Model 917 multichannel analyser (Roche Diagnostics, Indianapolis, IN) from 1999 to 2001 and a Beckman Synchron LX20 (Beckman Coulter, Inc., Brea, CA) in 2002. Distributions of UA results from the two laboratories were compared at the time of transition, and no significant differences were observed. Total BMD was measured by dual-energy X-ray absorptiometry.

In addition, the following covariates were included: age, sex, race/ethnicity, BMI, income-poverty ratio, physical activity, blood urea nitrogen, total protein, total cholesterol, serum phosphorus, and serum calcium. Details of sUA and total BMD measurement process and other covariate acquisition process are available at www.cdc.gov/nchs/nhanes/.

### Statistical Analyses

All estimates were calculated accounting for NHANES sample weights. Following adjustment for potential confounders, weighted multiple regression analyses were applied to estimate the independent relationship between sUA and total BMD. Weighted generalized additive models and smooth curve fittings were employed to address the non-linearity of sUA and total BMD in the subgroup analyses. After adjusting for the same covariates in the linear regression models, two-piecewise linear regression models were further applied to examine the threshold effect of sUA on total BMD.

Categorical variables were expressed as frequency or percentage. Continuous variables were expressed as means ± standard deviation. Weighted linear regression models (continuous variables) and weighted chi-square tests (categorical variables) were performed to calculate differences between different groups. *P* < 0.05 was considered statistically significant. All analyses were performed with Empower software (www.empowerstats.com; X&Y solutions, Inc., Boston MA) and R version 3.4.3 (http://www.R-project.org, The R Foundation).

## Results

Table 1 shows the description of weighted sociodemographic and medical characteristics of the participants. A total of 7,320 participants were included in this study. Of these participants, 56.91% were male, 62.22% were Whites, 14.33% were Blacks, and 11.03% were Mexican Americans. Among different groups of sUA (quartiles, Q1–Q4), age, sex, race/ethnicity, BMI, income–poverty ratio, physical activity, blood urea nitrogen, total protein, total cholesterol, serum phosphorus, serum calcium, and total BMD are all significantly different.

**Table 1:** Description of 7,320 participants included in the present study.

**Serum uric acid**	**All**	**Q1**	**Q2**	**Q3**	**Q4**	***P*-value**
Age (years)	15.50 ± 2.29	15.03 ± 2.38	15.29 ± 2.34	15.52 ± 2.24	16.04 ± 2.11	<0.0001
Sex (%)						<0.0001
Male	56.91	24.63	38.80	66.45	88.75	
Female	43.09	75.37	61.20	33.55	11.25	
Race/ethnicity (%)						<0.0001
White	62.22	54.98	61.83	65.86	64.83	
Black	14.33	18.37	15.34	13.97	10.64	
Mexican American	11.03	12.96	10.47	10.12	10.85	
Other	12.41	13.69	12.36	10.05	13.69	
BMI (kg/m^2^)	23.40 ± 5.61	21.12 ± 4.29	22.52 ± 5.02	23.37 ± 5.05	25.98 ± 6.42	<0.0001
Income poverty ratio	2.55 ± 1.62	2.45 ± 1.62	2.50 ± 1.60	2.65 ± 1.64	2.58 ± 1.63	0.0033
Physical activity (%)						<0.0001
Not walk very much	4.62	5.27	4.43	3.97	4.87	
Walk a lot	9.59	8.66	11.27	8.02	10.40	
Climb often	7.34	8.35	6.46	6.90	7.72	
Heavy activity	20.05	14.53	17.52	21.73	24.99	
Not recorded	58.39	63.20	60.32	59.38	52.03	
Blood urea nitrogen (mg/dL)	10.68 ± 3.29	9.86 ± 3.05	10.05 ± 3.15	10.93 ± 3.29	11.61 ± 3.34	<0.0001
Total protein (mg/dL)	7.36 ± 0.44	7.28 ± 0.46	7.30 ± 0.43	7.37 ± 0.43	7.47 ± 0.44	<0.0001
Total cholesterol (mg/dL)	161.55 ± 31.06	160.87 ± 28.46	159.52 ± 29.27	159.77 ± 31.94	165.47 ± 33.28	<0.0001
Serum phosphorus (mg/dL)	4.37 ± 0.68	4.43 ± 0.64	4.40 ± 0.68	4.40 ± 0.70	4.27 ± 0.70	<0.0001
Serum calcium (mg/dL)	9.72 ± 0.32	9.64 ± 0.32	9.69 ± 0.33	9.75 ± 0.31	9.79 ± 0.32	<0.0001
Total BMD (g/cm^2^)	1.08 ± 0.13	1.03 ± 0.11	1.06 ± 0.12	1.09 ± 0.13	1.12 ± 0.13	<0.0001
12–15 years	1.01 ± 0.11	0.98 ± 0.11	1.00 ± 0.10	1.02 ± 0.11	1.04 ± 0.10	<0.0001
16–19 years	1.14 ± 0.11	1.09 ± 0.09	1.12 ± 0.10	1.15 ± 0.11	1.17 ± 0.11	<0.0001

*Mean ± SD for continuous variables: P-value was calculated by weighted linear regression model*.

*% for categorical variables: P-value was calculated by weighted chi-square test*.

### Association Between sUA and Total BMD

**Figure 1:**
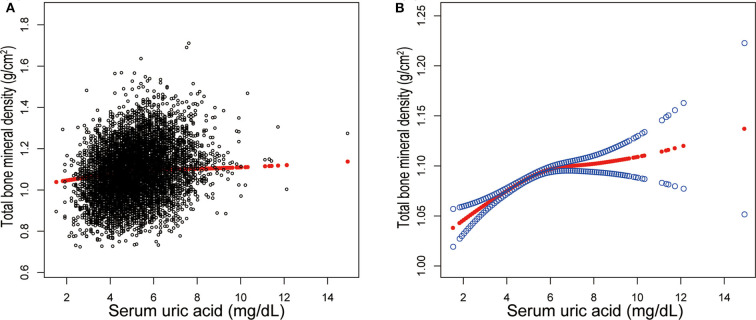
Correlation between serum uric acid and total bone mineral density. **(A)** Each black point represents a sample. **(B)** The area between two blue dotted lined is expressed as a 95% CI. Each point shows the magnitude of the serum uric acid and is connected to form a continuous line. Age, sex, race/ethnicity, body mass index, income poverty ratio, physical activity, blood urea nitrogen, total protein, total cholesterol, serum phosphorus, serum calcium were adjusted.

Three weighted univariate and multivariate linear regression models were constructed: model 1, not adjusted; model 2, age, sex, race/ethnicity were adjusted; model 3, the covariates presented in Table 1 were adjusted. In the fully-adjusted model, we observed a positive association between sUA and total BMD [0.0076 (0.0056, 0.0097)] (Table 2, Figure 1). However, when stratifying by age, sex, or race/ethnicity, this association was not significant in female adolescents [0.0014 (−0.0020, 0.0047)], adolescents aged 16–19 years [−0.0002 (−0.0032, 0.0028)], and other race/ethnicity [0.0038 (−0.0030, 0.0106)].

**Table 2:** Association of serum uric acid with total bone mineral density.

	**Model 1 β (95% CI)**	**Model 2 β (95% CI)**	**Model 3 β (95% CI)**
Serum uric acid	0.0195 (0.0176, 0.0213)	0.0164 (0.0144, 0.0184)	0.0076 (0.0056, 0.0097)
**Stratified by age**			
12–15 years	0.0171 (0.0143, 0.0198)	0.0157 (0.0131, 0.0182)	0.0074 (0.0048, 0.0101)
16–19 years	0.0216 (0.0191, 0.0241)	0.0084 (0.0055, 0.0112)	−0.0002 (−0.0032, 0.0028)
**Stratified by sex**			
Male	0.0341 (0.0309, 0.0373)	0.0142 (0.0117, 0.0166)	0.0057 (0.0031, 0.0082)
Female	0.0104 (0.0066, 0.0141)	0.0100 (0.0068, 0.0132)	0.0014 (−0.0020, 0.0047)
**Stratified by race/ethnicity**			
Whites	0.0286 (0.0245, 0.0327)	0.0153 (0.0114, 0.0191)	0.0076 (0.0036, 0.0115)
Blacks	0.0363 (0.0321, 0.0404)	0.0227 (0.0189, 0.0266)	0.0124 (0.0084, 0.0165)
Mexican Americans	0.0246 (0.0211, 0.0282)	0.0189 (0.0154, 0.0225)	0.0075 (0.0037, 0.0112)
Other race/ethnicity	0.0197 (0.0129, 0.0264)	0.0126 (0.0062, 0.0190)	0.0038 (−0.0030, 0.0106)

*Model 1, no covariates were adjusted*.

*Model 2, age, sex, race/ethnicity were adjusted*.

*Model 3, age, sex, race/ethnicity, body mass index, income-poverty ratio, physical activity, blood urea nitrogen, total protein, total cholesterol, serum phosphorus, serum calcium were adjusted*.

*In the subgroup analysis stratified by sex or race/ethnicity, the model is not adjusted for the stratification variable itself*.

In the subgroup analysis (Table 3), sUA level was significantly associated with higher total BMD in all groups of adolescents aged 12–15 years (*P* for trend <0.001 for each). While in adolescents aged 16–19 years, this association did not reach statistical significance in other groups except in Black subjects (*P* for trend = 0.039). The strongest association was observed in 12 to 15-year-old White subjects and 12 to 15-year-old Black subjects, in whom each quartile of sUA was increased.

**Table 3:** Total bone mineral density by quartiles of serum uric acid, stratified by race/ethnicity and age.

**Quartiles of serum uric acid**	**Total bone mineral density g/cm**^****2****^ **(95% Confidence Interval)**			
	**Whites**	**Blacks**	**Mexican Americans**	**Other race/ethnicity**
**12–15 years**				
Lowest quartiles	0.984 (0.971, 0.996)	1.038 (1.028, 1.047)	0.971 (0.960, 0.982)	0.966 (0.943, 0.989)
2nd	1.002 (0.990, 1.013)	1.057 (1.047, 1.068)	0.995 (0.984, 1.006)	0.998 (0.977, 1.019)
3rd	1.013 (1.002, 1.025)	1.079 (1.068, 1.090)	1.010 (1.000, 1.021)	1.035 (1.012, 1.057)
4th	1.016 (1.002, 1.030)	1.086 (1.071, 1.102)	1.014 (1.002, 1.026)	1.022 (1.000, 1.043)
*P* for trend	<0.001	<0.001	<0.001	<0.001
**16–19 years**				
Lowest quartiles	1.134 (1.117, 1.151)	1.195 (1.181, 1.209)	1.107 (1.095, 1.120)	1.152 (1.125, 1.178)
2nd	1.136 (1.123, 1.149)	1.196 (1.184, 1.207)	1.115 (1.104, 1.125)	1.143 (1.120, 1.166)
3rd	1.134 (1.123, 1.145)	1.214 (1.203, 1.224)	1.113 (1.103, 1.123)	1.141 (1.118, 1.163)
4th	1.135 (1.124, 1.147)	1.213 (1.201, 1.225)	1.106 (1.096, 1.116)	1.116 (1.093, 1.138)
*P* for trend	0.972	0.039	0.784	0.076

*Age, sex, body mass index, income-poverty ratio, physical activity, blood urea nitrogen, total protein, total cholesterol, serum phosphorus, serum calcium were adjusted*.

**Figure 2:**
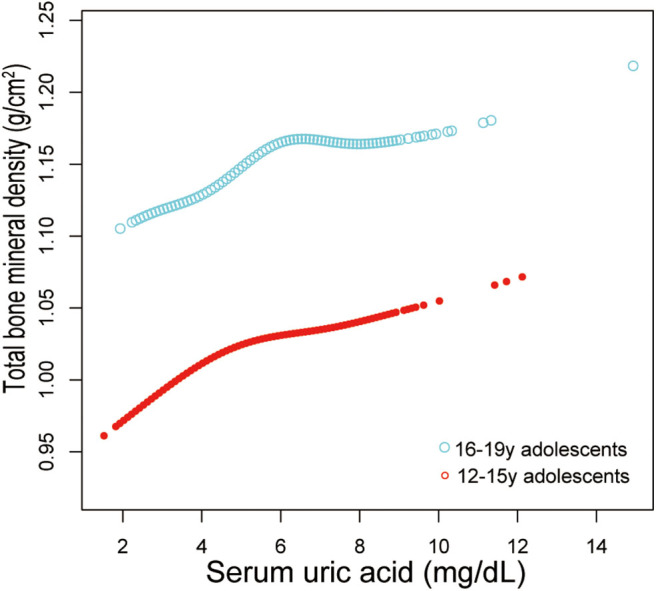
Serum uric acid and total bone mineral density dose–response relationship, stratified by age. Sex, race/ethnicity, body mass index, income poverty ratio, physical activity, blood urea nitrogen, total protein, total cholesterol, serum phosphorus, serum calcium were adjusted.

**Figure 3:**
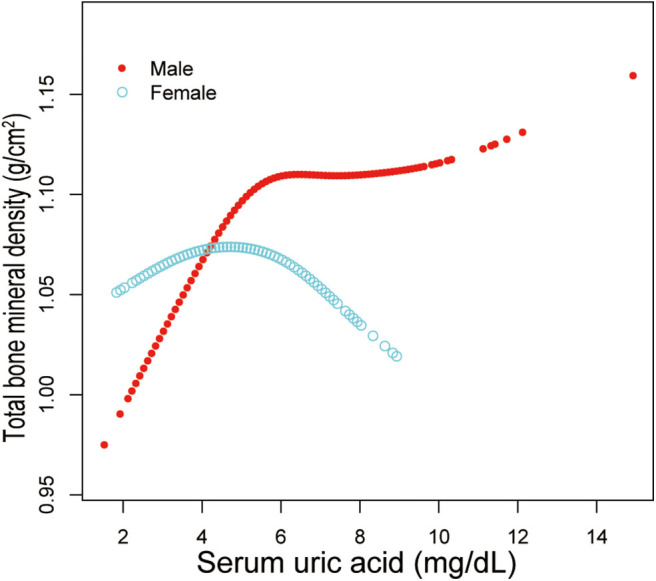
Serum uric acid and total bone mineral density dose–response relationship, stratified by sex. Age, race/ethnicity, body mass index, income poverty ratio, physical activity, blood urea nitrogen, total protein, total cholesterol, serum phosphorus, serum calcium were adjusted.

**Figure 4:**
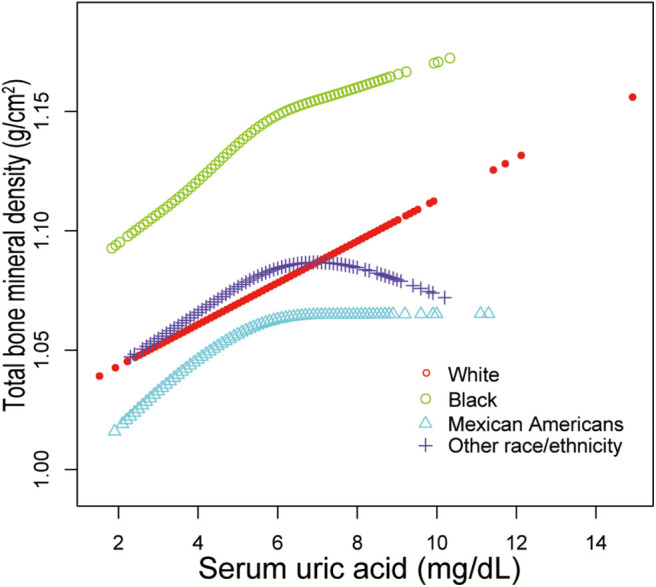
Serum uric acid and total bone mineral density dose–response relationship, stratified by race/ethnicity. Age, sex, body mass index, income poverty ratio, physical activity, blood urea nitrogen, total protein, total cholesterol, serum phosphorus, serum calcium were adjusted.

Additionally, adjusted smoothed plots suggested non-linear relationships between sUA and total BMD, stratified by age, sex, and race/ethnicity. Total BMD increased with sUA up to the turning point in adolescents aged 16–19 years (turning point: sUA 6.3 mg/dL) (Table 4, Figure 2). Likewise, there are turning points in female adolescents (turning point: sUA 3.9 mg/dL) (Table 4, Figure 3), and in other race/ethnicity (turning point: sUA 5.4 mg/dL) (Table 4, Figure 4). Taken together, the association between sUA and total BMD in female adolescents, adolescents aged 16–19 years, and other race/ethnicity followed an inverted U-shaped curve.

**Table 4:** Threshold effect analysis of serum uric acid on total bone mineral density using two-piecewise linear regression.

**Total bone mineral density**	**Adjusted ß (95% CI), *p*-value**
**16–19 years**	
Serum uric acid <6.3 (mg/dL)	0.0007 (−0.0035, 0.0049), 0.7446
Serum uric acid >6.3 (mg/dL)	−0.0017 (−0.0075, 0.0042), 0.5777
**Female**	
Serum uric acid <3.9 (mg/dL)	0.0245 (0.0143, 0.0347), <0.0001
Serum uric acid >3.9 (mg/dL)	−0.0054 (−0.0098, −0.0011), 0.0149
**Other race/ethnicity**	
Serum uric acid <5.4 (mg/dL)	0.0196 (0.0071, 0.0321), 0.0023
Serum uric acid >5.4 (mg/dL)	−0.0084 (−0.0191, 0.0023), 0.1254

*Age, sex, race/ethnicity, body mass index, income-poverty ratio, physical activity, blood urea nitrogen, total protein, total cholesterol, serum phosphorus, serum calcium were adjusted*.

*In the subgroup analysis for female and other race/ethnicity, the model is not adjusted for sex or race/ethnicity, respectively*.

## Discussion

The main objective of this study was to investigate whether sUA is independently associated with total BMD. In this study, we used a large and nationally representative sample of US adolescents. Our results suggested that sUA was positively associated with total BMD in male adolescents, adolescents aged 12–15 years, and White, Black and Mexican American adolescents, but not significant in female adolescents, adolescents aged 16–19 years, and other race/ethnicity.

The prevalence of hyperuricemia in general populations is relatively high and has been increasing over the past few decades (21, 22). Hyperuricemia also occurs at a younger age because of a combination of several factors, such as rapid urbanization, lifestyle changes, and obesity (23). Hyperuricemia is a key causal factor of gout, and all patients with gout are expected to experience at least some periods of hyperuricemia (24). However, gout is extremely rare in adolescents, and most patients with gout have an underlying disease. Recently, several epidemiological and laboratory studies discovered that UA is potentially involved in multiple biological processes and associated with a wide range of conditions, such as obesity, hypertension, and chronic kidney disease (25, 26). Hyperuricemia in adolescents is also a target of treatment. Our results supported that increased sUA level would be beneficial to bone health in adolescents with low sUA levels. Therefore, there is a need to understand the potential effects of sUA on bone health and balance potential risks against potential benefits. Thus, overcorrection of sUA level should be reconsidered.

Previous studies have examined the relationship between sUA and bone health. Their favorable association was reported in middle-aged or older Chinese population (9, 13–15) and other Asian population (10–12, 27, 28), but not in some Western population (29–31). An NHANES study conducted by Zhang et al. (32) reported no significant association between sUA level and BMD in participants aged >30 years. Their data also showed no difference in BMD between hyperuricemic rats and controls. Moreover, a Mendelian randomization study did not support a causal association between sUA and BMD (33). These conflicting conclusions may be attributed to the variations among these studies, in terms of demographic characteristics, study designs, study size, controlled confounders, etc.

Evidence linking sUA and BMD in adolescents is very limited. In a recent cross-sectional study of 413 Iranian adolescents aged 9–19 years, Karimi et al. (16) found a significant association between sUA and BMD by multiple regression analyses. Their results showed that those adolescents with greater BMD had higher sUA levels. The findings of our analysis would be consistent with this literature if we did not perform the subgroup analyses. Following the STROBE guideline (34), we performed subgroup analyses to make better use of the data. As a result, we found that this association was not significant in female adolescents, adolescents aged 16–19 years, and other race/ethnicity in our subgroup analyses. We also observed significant turning points in these groups. Their total BMD decreased when the sUA level was beyond those turning points. However, further large-sample prospective studies are needed to confirm this conclusion.

The exact mechanism of this association between sUA and bone metabolism remains unclear. A possible explanation to support the potentially beneficial effect of sUA on bone health might be related to its antioxidant capacity. Protective effect of UA on bone mass is supposed to be mediated through the free radical scavenging capacity during metabolic stress (35). Therefore, sUA may be involved in the pathogenesis of abnormal bone metabolism. UA was reported to decrease osteoclastogenesis in a dose-dependent manner and reduce the production of reactive oxygen species in osteoclast precursors (28). Another study showed that UA promoted proliferation of human bone mesenchymal stem cells and differentiation into osteoblasts and inhibited their adipogenic differentiation (36). There are other possible explanation for the association between sUA and bone health. Beneficial UA-BMD association might be partly mediated by muscle mass, similar to mechanical loading and muscle-derived cytokines (19). Despite these possibilities, further research is needed to explore the molecular mechanism toward the correlation between sUA and BMD, given the extreme importance of evidence to determine whether sUA concentration is an eligible diagnostic indicator for related diseases.

In this study, we analyzed representative samples of multiracial populations for better generalizability of the US population. Furthermore, this large sample size allowed us to perform further subgroup analyses. This is the main strength of this study. However, the limitations are worth noting. First, because this study has a cross-sectional design, it is difficult to determine whether there was a causal relationship between sUA and total BMD. Second, other confounding factors that were not included in this study may have an effect on the results. For example, sUA levels in adolescent boys are physiologically higher than those in girls. Studies have shown that this physiological difference was partially caused by the action of sex hormones on the renal excretion of UA (37). Thus, differences in sex hormones during pubertal development in adolescents aged 12–15 and 16–19 years might be a potential confounder to consider. Therefore, the role of UA in bone metabolism requires further clarification, and a longitudinal follow-up study with a large sample size will be needed.

In conclusion, this study demonstrated the correlation between sUA level and total BMD differed by sex. The increased sUA level would be beneficial to bone health in adolescents with low sUA levels, but for female adolescents, a higher sUA level (turning point, 3.9 mg/dL) may have an adverse effect on bone health. Further basic studies are needed to explore the effect of UA on bone health and the threshold sUA dose that is beneficial for bone health without deleterious effects.

## Data Availability Statement

The datasets for this study can be found at www.cdc.gov/nchs/nhanes/.

## Ethics Statement

The NCHS Ethics Review Board granted approval for the conduct of NHANES and written informed consents were obtained from all participants.

## Author Contributions

KP: writing—original draft preparation. XY and ML: validation. ZZ: writing—review and editing.

## Conflict of Interest

The authors declare that the research was conducted in the absence of any commercial or financial relationships that could be construed as a potential conflict of interest.
